# Increased Pro-Thrombotic Platelet Activity Associated with Thrombin/PAR1-Dependent Pathway Disorder in Patients with Secondary Progressive Multiple Sclerosis

**DOI:** 10.3390/ijms21207722

**Published:** 2020-10-19

**Authors:** Angela Dziedzic, Elzbieta Miller, Michal Bijak, Lukasz Przyslo, Joanna Saluk-Bijak

**Affiliations:** 1 Department of General Biochemistry, Faculty of Biology and Environmental Protection, University of Lodz, Pomorska 141/143, 90-236 Lodz, Poland; angela.dziedzic@unilodz.eu; 2 Department of Neurological Rehabilitation, Medical University of Lodz, Milionowa 14, 93-113 Lodz, Poland; elzbieta.dorota.miller@umed.lodz.pl; 3 Biohazard Prevention Centre, Faculty of Biology and Environmental Protection, University of Lodz, Pomorska 141/143, 90-236 Lodz, Poland; michal.bijak@biol.uni.lodz.pl; 4 Department of Developmental Neurology and Epileptology, Research Institute of Polish Mother’s Memorial Hospital, Rzgowska 281/289, 93-338 Lodz, Poland; lukasz.przyslo@iczmp.edu.pl

**Keywords:** thromboembolic consequences in multiple sclerosis, protease-activated receptors, blood platelets, megakaryocytes

## Abstract

Epidemiological studies confirm the high risk of ischemic events in multiple sclerosis (MS) that are associated with increased pro-thrombotic activity of blood platelets. The most potent physiological platelet agonist is thrombin, which activates platelets via cleavage of specific protease-activated receptors (PARs). Our current study is aimed to determine the potential genetics and proteomic abnormalities of PAR1 in both platelets and megakaryocytes, which may have thromboembolic consequences in the course of MS. The obtained results were correlated with the expression level of platelet and megakaryocyte transcripts for *APOA1* and *A2M* genes encoding atherosclerosis biomarkers: apolipoprotein A1 (ApoA1) and α-2-macroglobulin (α2M), respectively. Moreover, PAR1 functionality in MS platelets was assessed by flow cytometry, determining the level of platelet–platelet and platelet–leukocyte aggregates, platelet microparticles and surface expression of P-selectin. As a PAR1 agonist, the synthetic TRAP-6 peptide was used, which made it possible to achieve platelet activation in whole blood without triggering clotting. Comparative analyses showed an elevated level of platelet activation markers in the blood of MS patients compared to controls. The mRNA expression of gene coding α2M was upregulated, whilst ApoA1 was down-regulated, both in platelets and megakaryocytes from MS patients. Furthermore, we observed an increase in both mRNA expression and surface density of PAR1 in platelets and megakaryocytes in MS compared to controls. Both the level of platelet activation markers and PAR1 expression showed a high correlation with the expression of transcripts for *APOA1* and *A2M* genes.

## 1. Introduction

Multiple sclerosis (MS) is a major human autoimmune and degenerative disease of the central nervous system (CNS) with a variety of pathomechanisms making it a highly heterogeneous disease, considered a conglomerate of neurological syndromes [1]. Although MS is a neuroinflammatory disease of CNS, it is closely related to the damage of intracerebral blood vessels, mainly as a result of increased blood-brain barrier (BBB) permeability, and/or as a consequence of vessel occlusion [2]. The latest classification of MS clinical courses proposed in 2014 by Lublin et al. distinguishes two forms of the disease: initial MS, appearing as relapsing-remitting MS (clinically isolated syndrome (CIS) and RR MS patients); and progressive MS (primary progressive PP MS and secondary progressive SP MS patients), each one may be either in active or inactive forms [3].

Epidemiological studies have proved that comorbidities such as ischemic stroke, atherosclerosis, thrombosis, and myocardial infarction have been associated with increased mortality in patients with MS, especially in the progressive stage of MS [4,5,6]. The risk of death due to ischemic events was significantly greater (over 30%) in MS compared to the global age-matched population [7,8]. However, there is still a lack of research to explain this phenomenon. Recent meta-analyses and cohort studies concluded that MS is declared to be associated with cardiovascular disease (CVD) due to both thrombin upregulation and blood platelet pro-thrombotic over-activity [9,10,11]. Nevertheless, the vast majority of studies that confirmed the platelet abnormalities in MS patients referred only to the initial phase, that is RR MS [12,13,14]. The chronic activation of blood platelets in SP MS, as expressed by high levels of activation markers, altered mitochondrial function, and increased platelet reactivity, has been proven in our previous studies to focus exclusively on the progressive stage of MS, characterized with an especially high risk of CVD [15,16,17]. Furthermore, our findings have demonstrated changes in the protein profile, manifesting an enhanced level of blood coagulation factors [18].

An enhanced plasma potential to generate thrombin has been linked to an increased risk of venous thromboembolism due to promoting clot formation. Although the main function of thrombin is the conversion of fibrinogen to fibrin, this enzyme also exerts multiple cellular effects and can activate platelets and further augment inflammation via proteinase-activated receptors (PARs). The function of PAR1 and PAR4 has been well documented in human platelets [19,20]. PAR1 accounts for the initial platelet aggregation in response to thrombin, while PAR4 may contribute to the stability of platelet aggregation. PAR1 is a more sensitive receptor and is necessary for the fastest and most robust platelet responses, and PAR4 signaling is important when PAR1 function is disturbed. In contrast to PAR1, PAR4 mediates platelet activation only at high thrombin concentrations and PAR4 signaling appears unnecessary for platelet activation when PAR1 function is intact [21]. Thrombin is by far the most potent platelet agonist in vivo [22]. Moreover, it is documented that high concentration of thrombin may produce axonal damage and retraction, intracellular calcium upregulation, induce BBB damage, and finally, cell death [23]. It is considered that inhibition of PAR1 may reduce the activation of the coagulation pathway, enhance fibrinolysis and block the endothelium disruption in many inflammation diseases [24,25,26]. Furthermore, the latest research has demonstrated that there is a relationship between the occurrence of atherosclerosis and a decline in motor disability together with decreased cognitive function in MS patients [27]. Several biomarkers and atherosclerosis imaging measures have been evaluated for association with an incident of cardiovascular disease, including apolipoprotein A1 (ApoA1) and α-2-macroglobulin (α2M) [28,29].

The complexity of MS and its ambiguous etiology means that the current pharmacological treatments are only purposed at alleviating the course of the disease. The study on the development of antiplatelet therapies may provide benefits for patients with SP MS at high CVD risk. The studies scheduled by our research group direct the search for the origins of the pro-thrombotic potential of blood in SP MS towards molecular changes within blood platelets. Therefore, the scope of our current research is getting to know the molecular mechanisms of the heightened pro-thrombotic activity of blood platelets in SP MS, by genetic, proteomic, and functional analyses of their surface receptors. The knowledge gained so far allows us to assume that the platelet thrombin/PAR1 signaling pathway may be crucial for thromboembolic consequences observed in SP MS patients. This paper aims to verify this hypothesis. We have evaluated the expression of the *F2R* gene encoding the PAR1, and the concentration of the PAR1 molecules, both in blood platelets and megakaryocytes, which are platelet precursor cells. Our molecular results were correlated with gene expression of typical atherosclerotic markers such as α2M and ApoA1 measured also in platelets and megakaryocytes as transcripts for *APOA1* and *A2M* genes, respectively. Additionally, the functionality of the platelet PAR1 receptor was evaluated by determining platelet reactivity in response to the synthetic TRAP-6 peptide agonist that selectively interacts with PAR1. The use of TRAP-6 made it possible to assess the level of platelet reactivity using the cytometry method in whole blood without isolation of them from the physiological environment.

## 2. Results

### 2.1. The Level of Blood Platelet’s Markers of Activation

**Figure 1:**
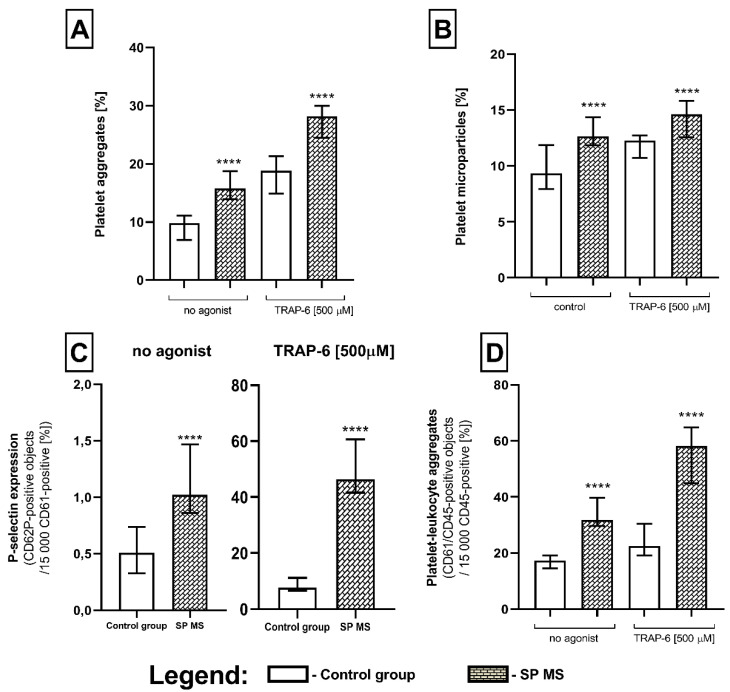
Platelet activation markers measured by double-labeled flow cytometry. The level of platelet aggregates (PAGs) (**A**) and platelet-derived microparticles (PMPs) (**B**) expressed as the percentage of platelet aggregates relative to the total platelet pool (15,000 CD61-positive objects). P-selectin expression level presented as the percentage of CD61/CD62-positive targets in the total platelet pool (15,000 CD61-positive targets) (**C**), as well as level of platelet-leukocyte aggregates (PLAs) relative to the total leukocyte pool (15,000 CD45-positive objects) (**D**). Statistical analysis was performed using the Mann–Whitney U test. The results are expressed as a median and interquartile range (IQR), **** *p* < 0.0001.

**Figure 2:**
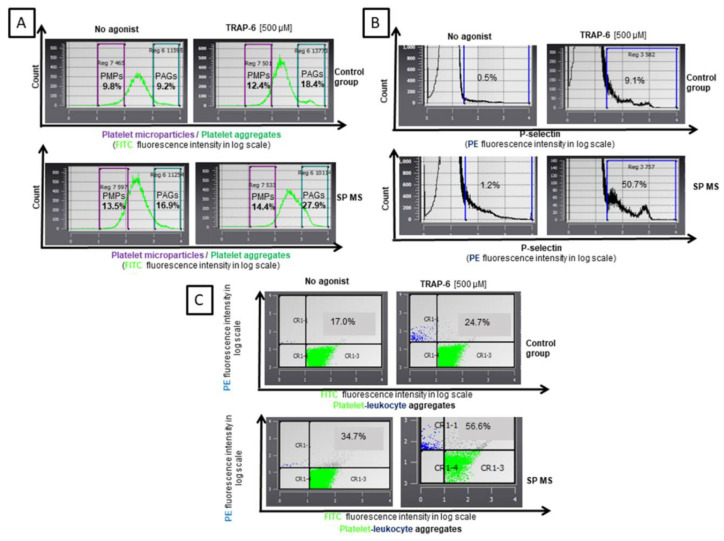
Representative histograms of platelet markers: %PMPs and %PAGs (**A**) and %P-selectin (**B**) and dot plots of the levels of %PLAs (**C**) in a population of resting platelets (no agonist) and in the population of TRAP-6-stimulated platelets, in whole blood samples obtained from secondary progressive (SP) multiple sclerosis (MS) patients and control group. The population of blood platelets were distinguished in whole blood based on the expression of CD61/FITC and labeled with monoclonal antibody CD62P/PE against the surface P-selectin or CD45/PE against leukocytes. All CD61/FITC-positive objects with a parameter forward scatter (FSC) below than 10^2^ were characterized as PMPs, above than 10^3^ as PAGs, while objects CD61/CD45-positive were recognized as PLAs. For each sample, 15,000 objects were acquired.

As a result of our analysis, we demonstrated an explicit increase in the formation of platelet aggregates (PAGs), platelet-derived microparticles (PMPs), platelet-leukocyte aggregates (PLAs), as well as a higher expression of surface P-selectin (CD62P) in SP MS patients compared to a control group, both in non-stimulated (no agonist) and TRAP-6-stimulated blood platelets. Whole blood assays performed by flow cytometry, without isolating platelets, avoided non-specific activation of these cells and the formation of artifacts. We showed that in quiescent platelets from SP MS patients there was an elevated level of PAGs (approximately 2-fold vs. control, *p* < 0.0001) (Figure 1A), PMPs (1.5-fold vs. control, *p* < 0.0001) (Figure 1B), PLAs (2-fold vs. control, *p* < 0.0001) (Figure 1C), as well as the higher surface expression of P-selectin (over 2-fold vs. control, *p* < 0.0001) (Figure 1D). The examination of blood platelets responsiveness to the action of TRAP-6 (500 µM) indicated a higher percentage of PAGs (1.5-fold vs. control, *p* < 0.0001) (Figure 1A), PMPs (1.2-fold vs. control, *p* < 0.0001) (Figure 1B), and PLAs (2-fold vs. control, *p* < 0.0001) (Figure 1C), and also a significantly increased expression of surface P-selectin in SP MS (approximately 6-fold increase, *p* < 0.0001) (Figure 1D). Figure 2 features the representative histograms, showing the percentage distribution of PAGs and PMPs in the total pool of CD61-positive objects (Figure 2A) and level of surface-exposed P-selectin (Figure 2B), as well as double fluorescence dot plots expressing the formation of PLAs (Figure 2C) in blood samples (without agonist and with TRAP-6) from the control group and SP MS.

### 2.2. The Expression for PAR1 at Protein and mRNA Level in Blood Platelets and Megakaryocytes

**Figure 3:**
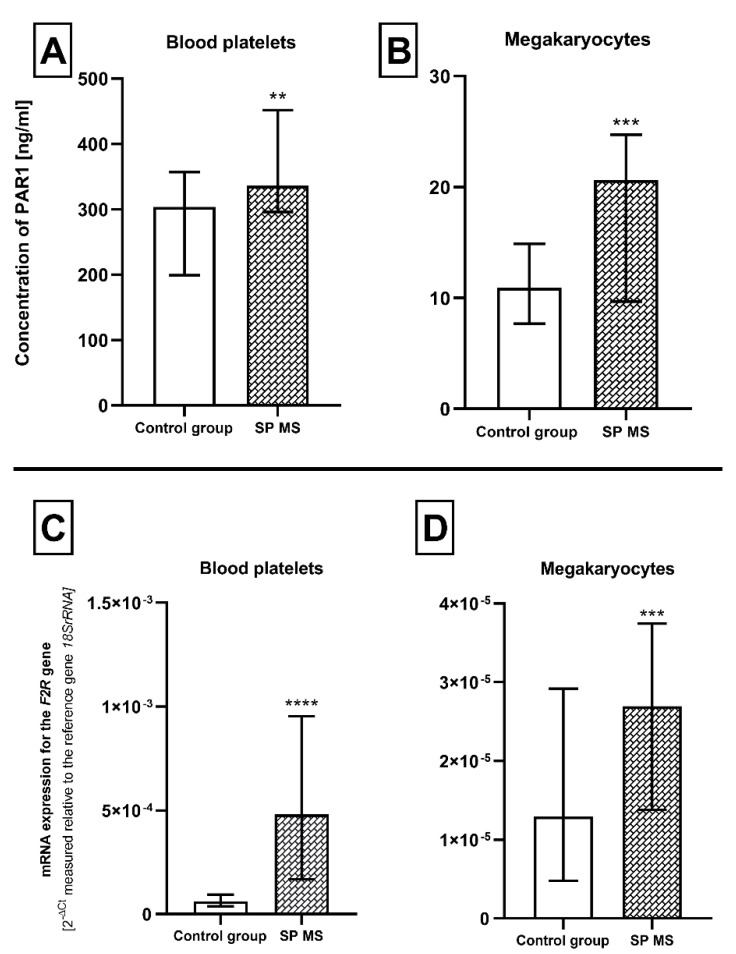
The level of PAR1 (ng/mL) ± SD and the relative expression of the *F2R* gene (according to the reference gene—*18SrRNA*) measured at the mRNA level in platelets (**A** and **C**, respectively) and megakaryocytes (**B** and **D**, respectively) from SP MS patients (*n* = 45) and the control group (*n* = 45). Statistical analysis was performed using the Mann–Whitney U test. The results are expressed as a median and interquartile range (IQR), ** *p* < 0.01, *** *p* < 0.001, **** *p* < 0.0001.

Our results indicated a significant increase in PAR1 expression in SP MS patients at the protein and mRNA level, both in platelets and megakaryocytes. Based on the results obtained by the ELISA method, we found that the average concentration of PAR1 molecules in platelet lysates from patients with SP MS (373 ± 115 ng/mL) was about 30% higher than the concentration in control platelets (268 ± 108 ng/mL) (*p* < 0.01) (Figure 3A). In the case of megakaryocytes, the mean concentration of PAR1 molecules was even 2-fold higher in SP MS as compared to the concentration in control (22.7 ± 16.9 vs. 10.7 ± 5.1 ng/mL, respectively) (*p* = 0.0008) (Figure 3B). The relative expression of mRNA transcripts for the *F2R* gene in blood platelets obtained from patients with SP MS was increased approximately 6-fold (*p* < 0.001) in comparison to the control group (Figure 3C). Simultaneously, the expression of this transcript in megakaryocytes increased by 40% compared to the control group (*p* < 0.001) (Figure 3D).

### 2.3. The Relative mRNA Expression of Genes Encoding Atherosclerosis Biomarkers—APOA1 and A2M in Blood Platelets and Megakaryocytes

**Figure 4:**
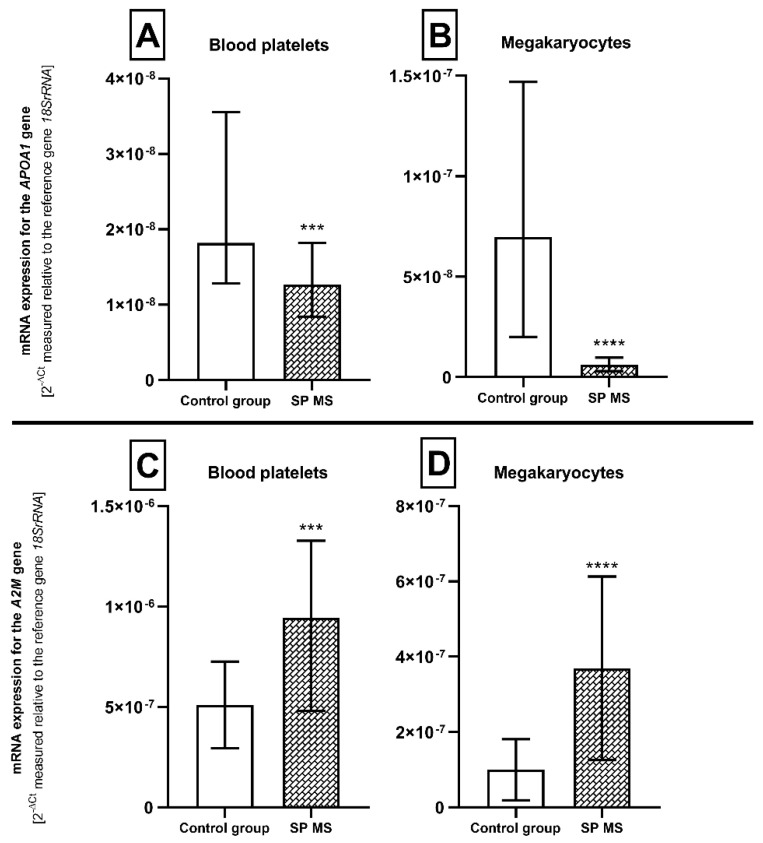
Expression of the *APOA1* and *A2M* genes (measured at the mRNA level) in platelets (**A** and **C**, respectively) and megakaryocytes (**B** and **D**, respectively) from SP MS patients (*n* = 45) and the control group (*n* = 45). Statistical analysis was performed using the Mann–Whitney U test. The results are expressed as the median of 2^−ΔCt^ and interquartile range (IQR) (according to the reference gene—*18SrRNA*), *** *p* < 0.001, **** *p* < 0.0001.

In the next set of experiments, we reported that the mRNA level of *APOA1* transcript was significantly lower both in platelets (approximately 1.5-fold) and megakaryocytes (near 12-fold) from SP MS patients compared to the control group (*p* < 0.001; *p* < 0.0001, respectively) (Figure 4A,B). In contrast, analysis of the relative expression of mRNA for the *A2M* gene in blood platelets showed more than a 2-fold statistically significant (*p* < 0.001) increase in SP MS (Figure 4C), compared to the control group. Analogically, in megakaryocytes the relative expression of mRNA for the *A2M* gene showed more than an approximately 4-fold statistically significant (*p* < 0.0001) increase in SP MS, compared to the control group (Figure 4D). Furthermore, Table 1 shows the calculated correlation between the expression level of transcripts for *APOA1* and *A2M* genes vs. parameters of platelet activity (PAGs, PMPs, P-selectin and PLAs) and PAR1 expression level.

## 3. Discussion

The role of platelets in mediating immune responses is becoming increasingly recognized [30], with the activation-induced up-regulation of a large receptor and molecule repertoire allowing platelets to interact with almost every type of immune cell [31]. The interaction of platelets with endothelial cells and inflammatory cells promotes leukocyte recruitment to the inflamed tissue [32]. The damage and increase of the BBB permeability facilitate the recruitment of inflammatory effector cells such as mononuclear cells and macrophages, and the activation of resident inflammatory microglial cells that promote the development of CNS lesions [33,34]. Blood platelets can directly activate leukocytes through a receptor-dependent mechanism or, indirectly, by biologically active compounds secreted from their granules. Activated platelets support leukocyte recruitment via formation of PLAs. Cell–cell interactions (PGAs and PLAs) provide critical mechanisms by which platelets link thrombosis, inflammation and related processes, such as diapedesis and leukocyte infiltration, which are critical in MS pathology [35]. Platelet–leukocyte interactions occur as a consequence of platelet activation and play a crucial role in the deposition of activated platelets in demyelinating lesions in MS, which leads to brain neurodegeneration [14]. Relatively recent proteomic analysis of chronic active MS brain lesions has revealed the crosstalk between coagulation, platelets and brain inflammation, and suggest that targeting platelet receptors may represent a novel attractive therapeutic approach to stifle the inflammatory response in MS. The presence of platelets in the chronic, active demyelinating MS lesions has been discovered. Simultaneously, the coagulation cascade, leading to the generation of large amounts of thrombin and responsible for platelet activation, was characterized as pivotal in terms of implications in MS brain lesion development [36,37]. Thrombin, also known as an active plasma coagulation factor II, belongs to the family of serine proteases and plays a crucial role in the blood coagulation process due to the conversion of soluble fibrinogen into insoluble fibrin, constituting a clot, and then the stabilization of the clot by transglutaminase factor XIII activation [38,39]. Moreover, even small amounts of thrombin generated in the coagulation pathway activate platelets and stimulate back other plasma coagulation factors (FXI, FVIII, FV) on the surface of activated platelets [40]. The blood platelets are the main elements of the cellular hemostasis but also play an important role in the coagulation cascade. Thrombin is the most potent platelet agonist and platelet responses to thrombin are primarily mediated through G-protein-coupled protease-activated receptors (GPCRs). PAR1 and PAR4 are present on human platelets (activated by the hexa-peptide sequence SFLLRN and GYPGQV, respectively), where PAR1 is the primary mediator of thrombin-stimulated platelet pro-thrombotic activity [41].

The significant role of thrombin in processes of hemostasis and thrombosis is associated with cardiovascular disturbances. Evidence of an increased effect of thrombin in vivo has been found in individuals at high risk of developing clinically significant thromboembolism in both the veins and arteries [42]. The reports related to vascular disease in MS, confirm an increased risk of ischemic events associated with abnormal coagulation cascade and an over-activity of platelets, especially ischemic stroke, myocardial infarction and venous thromboembolism [4,5,6,7,8,9,10,11,43]. A large Danish population-based cohort study revealed that death caused by vascular or cardiac diseases (mainly stroke and deep vein thrombosis) was the most frequently listed reason for death in MS. The increased risk was most pronounced during the first year following the initial MS diagnosis. However, also in long-term neurologically debilitated MS patients, immobilization is surely an important factor leading to increased risk of thromboembolism, even after controlling for traditional risk factors [44]. Treatments for MS may also increase the risk for CVD. There is a well-established association between the use of disease modifying therapies (DMTs) and higher CVD risk in MS [45,46]. In addition, the related disorders, such as atherogenic dyslipidemia, hypertension, or diabetes influence the risk of thromboembolism. MS and type I diabetes mellitus are autoimmune disorders, considered to be a closely related disease, where the presence of one of these diseases increases the tendency to develop the other. While hypertension, obesity and type 2 diabetes are common comorbidities, associated with a sedentary lifestyle [47]. Diabetes is associated with vascular disease. Chronic hyperglycemia and insulin resistance play an important role in the initiation of vascular complications, and involve a number of mechanisms including formation of glycation end products, oxidative stress, and inflammation. These conditions favor vascular endothelial damage and disturb hemostasis processes [48]. However, there is still little known about the mechanisms of cardiovascular dysfunction in MS, and the exact pathways to higher risk of CVD in MS are not yet completely explained. Given the crucial role ascribed to thrombin in the development of both venous and arterial thrombosis, in the presented work we undertook research aimed at determining the role of the thrombin/ PAR1-dependent pathway in increased pro-thrombotic platelet activity noticed in patients with SP MS. In this study, the activation of human platelets with PAR1-specific peptide agonist TRAP-6 has been investigated. The addition of TRAP-6 to platelets elicits the very strong activation response analogical to thrombin activation without the complications of fibrinogen cleavage and clot formation [30].

The first stage of our research tended to estimate the level of platelet activation parameters using the flow cytometry method. The basal level of typical markers of platelet activation was rated in the whole blood immediately after blood collection, reflecting the activation state of circulating platelets. At the same time, the application of TRAP-6 as an external agonist was to verify the functionality of the PAR1-dependent signal pathway by determining the reactivity of platelets ex vivo in their physiological environment. Our results argue in favor of the significant higher pro-thrombotic potential of blood in SP MS (Figure 1 and Figure 2). We proved the elevated subpopulations of platelet aggregates and increased generation of PMPs (vesicular structures mainly produced during activation and cell death) in unstimulated blood. The importance of PMPs’ role in strengthening the coagulation cascade is well known. However, PMP produced in inflammatory events can influence immune responses [49]. The elevated level of PMPs have been detected in RR MS patients [21,30,35] and in other autoimmune diseases, such as rheumatoid arthritis (RA) and systemic lupus erythematosus (SLE), which have been associated with disease activity [50,51], as well as in Alzheimer’s disease. PMPs display a broad spectrum of bioactive substances and active receptors derived from platelets [52]. Moreover, PMPs reveal measurable levels of complement deposition on the cell membrane, which can attract and activate leukocytes by interacting with complement receptors expressed on the leukocyte surface [53]. Among the platelet proteins contained in PMPs are the matrix metalloproteinase (MMP), especially MMP-2 and MMP-9, which have been generally recognized as major participants in the disruption of BBB in MS, by enabling the migration of white blood cells to the CNS [54]. PMPs are generated in response to several strong platelet agonists, including thrombin [55]; but the individual capacity of PAR1 and PAR4 to induce PMPs formation has not been explored.

However, among our results it is particularly noteworthy that despite elevated levels of P-selectin and platelet–leukocyte complexes in the unstimulated blood of patients with SP MS, the levels of these markers increased significantly after TRAP-6 stimulation. Based on flow cytometric measurements, we have shown a 2-fold higher P-selectin expression on the non-stimulated platelet in SP MS patients in comparison to the control group, while in TRAP-6-stimulated platelets the increase was much higher (approximately 6-fold vs. control) (Figure 1C). P-selectin (CD62P) is a glycoprotein presented in α-granules of resting platelets. During activation platelets dynamically change their shape and release the contents of their granules, including P-selectin. CD62P allows platelet–leukocyte interactions via P-selectin glycoprotein ligand 1 (PSGL-1) presented on the leukocyte surface, mainly on neutrophils and monocytes [56]. The formation of PLAs might be an intermediate stage in the development of inflammation into the endothelium due to elevating the level of mediators released from both platelets and immune cells, which activate and recruit new cells. Blood platelets have been even indicated to be a key element mediating the adhesion of circulating lymphocytes to the endothelium, which ensures their recruitment, initiates diapedesis and infiltration of T reactive lymphocytes into the affected vessel. It should be noted that the mutual cellular interactions between platelets, immune cells and endothelium are closely related to activation of all types of these cells, which can have serious consequences for maintaining the tightness of BBB and further development of local neuroinflammatory response and demyelination. BBB permeability disorder is crucial for vascular consequences leading to the development of both cardiovascular diseases and MS development [57,58].

Despite the relatively well-documented role of platelets in the pathomechanisms of inflammation and neurodegeneration, and the increased activity of circulating platelets in MS, confirmed in clinical trials, there are still no studies to explain the molecular basis of impaired platelet function in this disease. Recognition of the genetic factors attributed to increased platelet activity, leading to the formation of blood clots and supporting coagulation cascade, can help to identify the molecular mechanisms of this phenomenon. In recent years, many studies have shown that, despite the lack of a cell nucleus, platelets are capable of synthesizing proteins, and the level of many of them is dependent on the degree of platelet activation [59]. Platelets contain about 5000 of the mRNA transcripts derived from megakaryocytes, the giant bone marrow cells, that are precursors of platelets [60].

In the next step of our experiments we verified the potential genetic and proteomic abnormalities of the platelet PAR1 receptor as a potential cause of the increased reactivity we observed in the cytometric analysis. In order to determine the molecular mechanisms of revealed phenotypic changes, the comparative analysis of the PAR1 expression level included the simultaneous assessment of the changes at mRNA level and protein concentration of this receptor molecule. Studies completed in recent years have shown that platelet proteins may have different origins: some of them are synthesized based on mRNA present in blood platelets but a certain amount of platelet proteins are synthesized in megakaryocytes and flow from mature megakaryocytes to the formed platelets [61,62]. Therefore, the genetic background of molecular changes of platelet proteome should be defined in both the platelets and megakaryocytes stage. For this reason, we conducted the multidirectional analysis focused on the assessment of the molecular changes at mRNA and protein levels in both platelets and megakaryocytes. For the first time, we found that platelet PAR1 expression (as mRNA for the *F2R* gene and protein molecules) is enhanced in SP MS patients (Figure 3) and we postulate that this phenomenon may be responsible for the enhanced platelet thrombin/PAR1 signaling pathway leading to platelet hyperactivity.

Damage to the endothelial layer of blood vessels and conditions that promote persistent inflammation within the endothelium pose a risk of developing atherosclerosis. However, until now, biomarkers of atherosclerosis have not been analyzed in people with MS. The currently available literature indicated only the elevated carotid intima-media thickness (CIMT) as a possible predisposing factor to subclinical atherosclerosis in MS [63]. However, further investigation in a large MS population is still needed. Hence, in our work, we decided to measure the level of recognized atherosclerosis biomarkers in blood platelets and megakaryocytes from SP MS patients and compare it with results from the control group. The main plasma risk factors that promote the development of atherosclerosis are elevated low-density lipoprotein (LDL) cholesterol level, low high-density lipoprotein (HDL) level, lipidogram disorders, as well as low level of ApoA1 [64]. One of the potential biomarkers of atherosclerosis analyzed in this work is ApoA1, the major protein constituent of the plasma HDL complex. Epidemiologic studies have confirmed that reduced levels of HDL and ApoA1 have a pro-atherogenic effect [65,66,67]. Our analysis showed for the first time that the relative expression of mRNA transcripts for the *APOA1* gene was significantly lower both in platelets and megakaryocytes from SP MS patients compared to the control group (Figure 4A,B). A low ApoA1 expression level indicates an increased risk of cardiovascular disease, especially in the presence of exaggerated pro-thrombotic features of blood platelets. Our studies find a high negative correlation between *APOA1* gene expression and TRAP-induced reactivity of blood platelets, as well as expression of PAR1 at the mRNA and protein level (Table 1). Our findings add to the accumulating evidence that changes in cholesterol pathway biomarkers are associated with progression of the neurodegenerative phase of the MS course. Murali et al. showed a decrease in ApoA1 level in patients transitioning into SP MS compared to the baseline level of ApoA1 in patients who remain RR MS. Percentage reduction in the ApoA1 level has been a significantly related to the number of new demyelinating lesions and gray matter loss in MS [68]. The neuroprotective properties of ApoA1 have also been confirmed in several other recent reports. These findings have provided evidence that higher level of ApoA1 is significantly correlated with better cerebral perfusion in MS patients and less gray matter and cortical atrophy after adjusting for the baseline serum neurofilament (sNfL) level as a marker of neurodegeneration [69,70,71].

In our previous work, analyzing proteome of platelets using a 2-dimensional electrophoresis (2-DE) method together with mass spectrometry (MALDI/TOF-TOF) analysis, we reported a higher level of α2M in SP MS patients compared to control group [18]. It is mainly produced by the liver, but also locally synthesized by blood cells. In human blood, its function is related to the regulation of the immune system, the control of the blood coagulation system and the suppression of the fibrinolytic system [72]. α2M is a highly abundant serum protein involved in the development of atherosclerosis; however, its circulating concentrations in human health/diseases are not exactly known [73]. The *A2M* gene expression modulates the severity and extent of atherosclerosis [74]. Therefore, α2-macroglobulin has been implicated as a potential marker for the diagnosis and prognosis of ischemic events [75,76]. Our analysis demonstrated an increased relative mRNA transcripts expression for the *A2M* gene both in platelets and megakaryocytes from SP MS patients compared to the control group (Figure 4C,D). According to the pro-thrombotic activity of platelets in MS, this finding seems to be particularly interesting due to its positive correlations with all platelet parameters measured above (Table 1). Our analysis showed for the first time that the relative mRNA transcripts expression for the *APOA1* gene was significantly lower both in platelets and megakaryocytes from SP MS patients compared to the control group (1.5-fold and 12-fold, respectively).

## 4. Materials and Methods

### 4.1. Clinical Characteristics of MS Patients and Control Group

Blood samples from SP MS patients and control group were collected into CPDA-1 (citrate phosphate dextrose adenine-1) tubes (Sarstedt^®^, Nümbrecht, Germany), in the morning (8–9 a.m.) in fasting status and stored under the same conditions, according to one established protocol. The study samples were obtained from 45 SP MS patients (Female *n* = 27; Male *n* = 18) diagnosed with SP MS. Patients were observed for one year and diagnosed according to the revised McDonald criteria, which were established by Lublin et al. [77]. The blood samples from SP MS patients were collected at the Neurological Rehabilitation Division III General Hospital in Lodz, Poland. Control blood samples were collected from 45 healthy volunteers (Female *n* = 28; Male *n* = 17) at Laboratory Diagnostics Center in Lodz, Poland. The control group was matched by age and gender. Healthy volunteers were not taking any medications, and they had never been diagnosed with MS or other chronic disease, coagulation disorders, any neurological or hormonal illnesses or any chronic inflammatory disease. Subjects enrolled in both groups receive no medicaments that could modulate platelet count, their function or may have influenced platelet activation or a clotting cascade. MS patients were not taking the DMTs for at least a year. Each healthy volunteer was screened (including hematological and biochemical tests) to rule out any medical conditions. The clinical parameters of the enrolled SP MS patients and controls are included in Table 2.

All patients were informed in sufficient detail about the merits, responsibilities, and confidentiality of a participant of the study. They voluntarily decided to participate, as well as acknowledged the possibility of subsequent resignation by signing the Patient’s Informed Consent Form. The protocol and all procedures were carried out according to the Helsinki Declaration and were approved by the Ethics Committee of the Faculty of Biology and Environmental Protection of University of Lodz, Poland, No. 15/KBBN-UŁ/II/2016.

### 4.2. Cytometric Measurement of Characteristic Markers of Platelet Activation

The level of typical markers of platelet activation: formation of AGs, PMPs, PLAs, as well the P-selectin surface expression were evaluated using flow cytometry analysis. The fresh whole blood samples, without the agonist or activated with TRAP-6 (500 µM, 10 min., 37 °C) (Sigma-Aldrich, St. Louis, MO, USA, were fixed in 1% CellFIX (Becton Dickinson, San Diego, CA, USA) solution for 1 h at 37 °C, and stained with anti-CD61/FITC, and additionally with anti-CD62P/PE or anti-CD45/PE antibodies (Becton Dickinson, San Diego, CA, USA) (30 min. in dark, 25 °C). Before analysis, the prepared samples were dissolved in 0.9% NaCl (20:1) and vortexed. Fluorescence of 15,000 events (CD61/FITC- or CD45/PE-positive objects (platelets and leukocytes, respectively) was measured each time. Gates for PE and FITC fluorescents were estimated, based on the fluorescence of unstained probes. Surface expression of P-selectin on CD61/FITC-positive cells was determined by fluorescence of CD62/PE. The percentage of CD62P-positive platelets was calculated relative to the total pool of platelets in each sample (15,000 CD61-positive objects), while the level of CD61/CD45-positive objects (PLAs) was calculated relative to the total pool of leukocytes (15,000 CD45-positive objects) measured in each sample. All data analyses were performed in Cube 6 flow cytometry with CyView Software v.1.5.5.8 (Partec, Goerlitz, Germany).

### 4.3. Isolation of Blood Platelets

Platelet-rich plasma (PRP) was obtained from whole blood by differential centrifugation (1200 rpmi, 12 min., 25 °C). To eliminate erythrocyte/leukocyte contamination, the PRP was purified using nano-sized MicroBeads conjugated to monoclonal human antibodies CD235a and CD45 (Miltenyi Biotec, Bergisch Gladbach, Germany), respectively. Subsequently, the PRP was loaded in the appropriate amount onto a MACS MS Column, which was placed in the magnetic field of a MiniMACS Separator. The magnetically labeled CD235a-positive and CD45-positive cells were retained on the column, and free-flowing purified blood platelets were collected in a sterile tube. The number of platelets was determined photometrically, and the final concentration of the platelet suspension was each time diluted to 2 × 10^8^ platelets/mL. The platelet suspension was divided into two equal portions, into one part the RNALater solution (Invitrogen, Carlsbad, CA, USA), and into the second part, the cell lysing buffer (2 M thiourea, 7 M urea, 4% CHAPS, 30 mM Tris; all reagents were purchased from Sigma-Aldrich, St. Louis, MO, USA) were added and immediately frozen (−80°C) for further transcriptomic and proteomic analysis.

### 4.4. Isolation of Megakaryocytes

First, the peripheral blood mononuclear cells (PBMCs) were isolated by density gradient centrifugation. Fresh whole blood was layered in portions (1:1 ratio) onto Gradisol G (Aqua-MED, Lodz, Poland), and centrifuged (2000 rpmi, 30 min., 25 °C). Subsequently, the buffy coat layer with PBMCs fraction was carefully collected, washed twice using PBS (pH 7.4) (Biosigma, Venice, Italy), and centrifuged (1600 rpmi, 12 min., 25 °C). Obtained PBMC sediment was suspended in reaction buffer (pH 7.4) (2 mM EDTA, 0.5% BSA, PBS pH 7.4). The second step was a magnetic separation, in which the CD61-positive PBMCs (megakaryocytes) were firstly labeled with anti-CD61 MicroBeads (Miltenyi Biotec, Bergisch Gladbach, Germany). Then, the cell suspension was loaded onto a MACS Column placed in the magnetic field of MACS Separator. The retained on the column megakaryocytes were flushed out together with applied reaction buffer by firmly pushing the plunger in the column. Finally, megakaryocyte suspension was divided into two equal portions and into one part the RNALater solution (Invitrogen, Carlsbad, CA, USA), and into the second part the cell lysing buffer (2 M thiourea, 7 M urea, 4% CHAPS, 30 mM Tris; all reagents were purchased from Sigma-Aldrich, St. Louis, MO, USA) were added, and samples were immediately frozen (−80 °C) for further transcriptomic and proteomic analysis.

### 4.5. Isolation of mRNA and cDNA Synthesis

Total RNA was isolated from frozen (−80 °C) blood platelets and megakaryocytes suspended in the RNALater solution using the ISOLATE II RNA Mini Kit (Bioline, London, UK). This kit contains a set of reagents that allows inactivation of RNases while stabilizing RNA molecules and the complete separation from DNA and proteins. Total RNA purity and concentration were measured by comparing the absorbance at 260 and 280 nm, respectively. Then, samples of total RNA were stored at −80 °C until used. The RT-PCR reaction was performed using the Maxima First Strand cDNA Synthesis Kit for RT-qPCR (Thermo Fisher Scientific, Waltham, MA, USA) following the manufacturer’s instructions.

### 4.6. Expression of the F2R, APOA1 and A2M Genes at the mRNA Level in Blood Platelets and Megakaryocytes

Quantitative Real-Time PCR (RT-qPCR) was performed to determine the relative expression of mRNA using the following TaqMan probes: *F2R* (Hs01881698_s1), *APOA1* (Hs00985000_g1), *A2M* (Hs00929971_m1). As an endogenous control, human *18SrRNA* (Hs99999901_s1) was used (Life Technologies, Carlsbad, CA, USA). Gene expression measurements were made on the Real-Time PCR—The CFX96TM Touch System (Bio-Rad, Herkules, CA, USA) using a TaqMan Universal Master Mix II, no UNG (Life Technologies, Carlsbad, CA, USA). The whole procedure was executed following the manufacturer’s protocol. To calculate the relative expression of studied genes, the equation 2^−ΔCt^ (ΔCt = Ct*_target gene_*− Ct*_18SrRNA_*) was used.

### 4.7. Concentration of PAR1 Receptor in Blood Platelets and Megakaryocytes

The level of the PAR1 receptor was measured in blood platelets and megakaryocytes using the commercial Human PAR1 ELISA Kit (Fine Test, Wuhan, China) following the manufacturer’s protocol. All measurements were made using MaxiSorp plates (Nunc, Roskilde, Denmark). Absorbance was measured at 450 nm using the SPECTROStar Nano Microplate Reader (BMG Labtech, Ortenberg, Germany). The level of PAR1 in blood platelet and megakaryocyte lysates was determined based on a standard curve expressed as ng/mL.

### 4.8. Statistical Analysis

The StatsDirect statistical software version V.2.7.2. was utilized for the statistical analysis. All values were expressed as a mean ± SD. The Shapiro–Wilk test was used to analyze the normality of the distribution of results. The significance of differences between the values was analyzed, depending on the normality, by unpaired t-Student test (for data with normal distribution) or Mann–Whitney U test (for data with abnormal distribution). Spearman’s rank correlation was used for interdependence analysis between atherosclerosis biomarkers and parameters: PAR1 expression and platelet activation markers. Multiple comparisons were performed using Benjamini–Hochberg, Holm–Sidak and Bonferroni–Sidak corrections. A level of *p* < 0.05 was accepted as statistically significant.

## 5. Conclusions

Understanding the complex pathomechanisms of MS is essential for improved therapies. Therefore, identification of targets specific to individual pathology in MS may have therapeutic benefits. Our lack of clinical data prevents us from suggesting guidelines for CVDs prophylaxis in MS patients. However, as CVD causes serious complications and still remains the leading cause of death, efforts to prevent CVD especially requires identification and targeting of high-risk MS patients. Considering the most probable ischemic events as a result of a coincidence of atherosclerosis risk factors and an increased PAR1 dependent activation pathway, we conclude that targeting platelet PAR1 may represent a novel therapeutic approach to the suppression of the adverse thrombotic consequences in MS.

## Figures and Tables

**Table 1:** Correlation coefficient value obtained for expression of atherosclerosis biomarkers (*A2M* and *APOA1*) and PAR1 expression and level of platelet activity markers (PAGs, PMPs, P-selectin and PLAs). The correlation was analyzed using Spearman’s rank correlation (Rho) method. The table consists of Rho, probability of correlation (*p*) and adjusted *p*-values for multiple tests by Benjamini–Hochberg, Holm–Sidak and Bonferroni–Sidak methods. Corrected *p*-values below 0.05 are considered as statistically significant and highlighted in bold.

Correlated Parameters	Corrections				
			Benjamini–Hochberg (Desired FDR (Q) = 5%)	Holm–Sidak (α = 0.05)	Bonferroni–Sidak (α = 0.05)
	Rho	*p*-Value	Adjusted *p*_1_-Value	Adjusted *p*_2_-Value	Adjusted *p*_3_-Value
mRNA Expression for the *A2M* gene in Control Blood Platelets vs.					
mRNA expression for the *PAR1* gene	0.5986	**0.0001**	**0.0003**	**0.001**	**0.001**
PAR1 (ng/mL)	0.6525	**0.0023**	**0.0038**	**0.0114**	**0.0228**
PAGs (%) (no agonist)	0.6028	**0.0005**	**0.001**	**0.003**	**0.005**
PAGs (%) (TRAP-6)	0.5251	**0.0032**	**0.0046**	**0.0127**	**0.0315**
PMPs (%) (no agonist)	0.3925	**0.0327**	**0.0327**	**0.0492**	0.2828
PMPs (%) (TRAP-6)	0.6741	**0.0001**	**0.0003**	**0.001**	**0.001**
P-selectin (%) (no agonist)	0.4104	**0.0249**	**0.0277**	**0.0492**	**0.2229**
P-selectin (%) (TRAP-6)	0.7294	**0.0001**	**0.0003**	**0.001**	**0.001**
PLAs (%) (no agonist)	0.4430	**0.015**	**0.0188**	**0.0443**	0.1403
PLAs (%) (TRAP-6)	0.6154	**0.0004**	**0.001**	**0.0028**	**0.004**
mRNA expression for the *A2M* gene in SP MS Blood Platelets vs.					
mRNA expression for the *PAR1* gene	0.8454	**0.0001**	**0.0003**	**0.001**	**0.001**
PAR1 (ng/mL)	0.5994	**0.0001**	**0.0003**	**0.001**	**0.001**
PAGs (%) (no agonist)	0.6628	**0.0001**	**0.0003**	**0.001**	**0.001**
PAGs (%) (TRAP-6)	0.4314	**0.018**	**0.02**	**0.0357**	0.1661
PMPs (%) (no agonist)	0.4003	**0.0291**	**0.0291**	**0.0357**	0.2557
PMPs (%) (TRAP-6)	0.4870	**0.0069**	**0.0099**	**0.0273**	0.0669
P-selectin (%) (no agonist)	0.6082	**0.0005**	**0.0008**	**0.0025**	**0.005**
P-selectin (%) (TRAP-6)	0.4724	**0.009**	**0.0113**	**0.0273**	0.0864
PLAs (%) (no agonist)	0.6367	**0.0002**	**0.0004**	**0.0012**	**0.002**
PLAs (%) (TRAP-6)	0.6935	**0.0001**	**0.0003**	**0.001**	**0.001**
mRNA expression for the *A2M* gene in Control Megakaryocytes vs.					
mRNA expression for the *PAR1* gene	0.4322	**0.0033**	**0.0039**	**0.0099**	**0.0325**
PAR1 (ng/mL)	0.6897	**0.002**	**0.0029**	**0.008**	**0.0198**
PAGs (%) (no agonist)	0.6175	**0.0005**	**0.0013**	**0.0035**	**0.005**
PAGs (%) (TRAP-6)	0.4474	**0.0138**	**0.0138**	**0.0138**	0.1297
PMPs (%) (no agonist)	0.5658	**0.0013**	**0.0023**	**0.0078**	**0.0129**
PMPs (%) (TRAP-6)	0.6806	**0.0001**	**0.001**	**0.001**	**0.001**
P-selectin (%) (no agonist)	0.5220	**0.0035**	**0.0039**	**0.0099**	**0.0345**
P-selectin (%) (TRAP-6)	0.6259	**0.0003**	**0.001**	**0.0027**	**0.003**
PLAs (%) (no agonist)	0.5639	**0.0014**	**0.0023**	**0.0078**	**0.0139**
PLAs (%) (TRAP-6)	0.6325	**0.0003**	**0.001**	**0.0027**	**0.003**
mRNA expression for the *A2M* gene in SP MS Megakaryocytes vs.					
mRNA expression for the *PAR1* gene	0.7112	**0.0006**	**0.0009**	**0.003**	**0.006**
PAR1 (ng/mL)	0.7112	**0.0006**	**0.0009**	**0.003**	**0.006**
PAGs (%) (no agonist)	0.6533	**0.0001**	**0.0003**	**0.001**	**0.001**
PAGs (%) (TRAP-6)	0.5111	**0.0043**	**0.0043**	**0.0074**	**0.0422**
PMPs (%) (no agonist)	0.7447	**0.0001**	**0.0003**	**0.001**	**0.001**
PMPs (%) (TRAP-6)	0.6565	**0.0001**	**0.0003**	**0.001**	**0.001**
P-selectin (%) (no agonist)	0.6110	**0.0004**	**0.0008**	**0.0028**	**0.004**
P-selectin (%) (TRAP-6)	0.6157	**0.0004**	**0.0008**	**0.0028**	**0.004**
PLAs (%) (no agonist)	0.5728	**0.0011**	**0.0014**	**0.0033**	**0.0109**
PLAs (%) (TRAP-6)	0.5193	**0.0037**	**0.0041**	**0.0074**	**0.0364**
mRNA expression for the *APOA1* gene in Control Blood Platelets vs.					
mRNA expression for the *PAR1* gene	−0.5779	**0.0001**	**0.0003**	**0.001**	**0.001**
PAR1 (ng/mL)	−0.4964	**0.0276**	**0.0276**	**0.0276**	0.2441
PAGs (%) (no agonist)	−0.7107	**0.0001**	**0.0003**	**0.001**	**0.001**
PAGs (%) (TRAP-6)	−0.7035	**0.0001**	**0.0003**	**0.001**	**0.001**
PMPs (%) (no agonist)	−0.4675	**0.0098**	**0.0109**	**0.0253**	0.0938
PMPs (%) (TRAP-6)	−0.6024	**0.0011**	**0.0024**	**0.0077**	**0.0109**
P-selectin (%) (no agonist)	−0.4835	**0.0085**	**0.0106**	**0.0253**	0.0818
P-selectin (%) (TRAP-6)	−0.5713	**0.0012**	**0.0024**	**0.0077**	**0.0119**
PLAs (%) (no agonist)	−0.5112	**0.0051**	**0.0073**	**0.0202**	**0.0498**
PLAs (%) (TRAP-6)	−0.5412	**0.0023**	**0.0038**	**0.0114**	**0.0228**
mRNA expression for the *APOA1* gene in SP MS Blood Platelets vs.					
mRNA expression for the *PAR1* gene	−0.4046	**0.0062**	**0.0089**	**0.0246**	0.0603
PAR1 (ng/mL)	−0.7693	**0.0001**	**0.0003**	**0.001**	**0.001**
PAGs (%) (no agonist)	−0.6823	**0.0001**	**0.0003**	**0.001**	**0.001**
PAGs (%) (TRAP-6)	−0.6809	**0.0001**	**0.0003**	**0.001**	**0.001**
PMPs (%) (no agonist)	−0.3627	**0.0495**	**0.0495**	**0.0495**	0.3981
PMPs (%) (TRAP-6)	−0.6714	**0.0001**	**0.0003**	**0.001**	**0.001**
P-selectin (%) (no agonist)	−0.4691	**0.0096**	**0.012**	**0.0285**	**0.092**
P-selectin (%) (TRAP-6)	−0.5538	**0.0018**	**0.0036**	**0.0108**	**0.0179**
PLAs (%) (no agonist)	−0.4546	**0.0123**	**0.0137**	**0.0285**	0.1164
PLAs (%) (TRAP-6)	−0.5292	**0.003**	**0.005**	**0.0149**	**0.0296**
mRNA expression for the *APOA1* gene in Control Megakaryocytes vs.					
mRNA expression for the *PAR1* gene	−0.4631	**0.0106**	**0.0151**	**0.0417**	0.1011
PAR1 (ng/mL)	−0.7127	**0.0006**	**0.0043**	**0.006**	**0.006**
PAGs (%) (no agonist)	−0.5555	**0.0017**	**0.0043**	**0.0119**	**0.0169**
PAGs (%) (TRAP-6)	−0.5604	**0.0015**	**0.0043**	**0.0119**	**0.0149**
PMPs (%) (no agonist)	−0.4843	**0.013**	**0.0163**	**0.0417**	0.1227
PMPs (%) (TRAP-6)	−0.5156	**0.004**	**0.008**	**0.0238**	**0.0393**
P-selectin (%) (no agonist)	−0.4129	**0.0241**	**0.0241**	**0.0417**	0.2165
P-selectin (%) (TRAP-6)	−0.5700	**0.0012**	**0.0043**	**0.0107**	**0.0119**
PLAs (%) (no agonist)	−0.4942	**0.006**	**0.01**	**0.0296**	0.0584
PLAs (%) (TRAP-6)	−0.4320	**0.0179**	**0.0199**	**0.0417**	0.1652
mRNA expression for the *APOA1* gene in SP MS Megakaryocytes vs.					
mRNA expression for the *PAR1* gene	−0.5478	**0.0024**	**0.008**	**0.0205**	**0.0237**
PAR1 (ng/mL)	−0.5737	**0.0092**	**0.0153**	**0.0452**	0.0883
PAGs (%) (no agonist)	−0.4983	**0.0065**	**0.013**	**0.0392**	0.0631
PAGs (%) (TRAP-6)	−0.3698	**0.049**	**0.049**	**0.0885**	0.3949
PMPs (%) (no agonist)	−0.5896	**0.0023**	**0.008**	**0.0205**	**0.0228**
PMPs (%) (TRAP-6)	−0.5887	**0.0057**	**0.013**	**0.0392**	0.0556
P-selectin (%) (no agonist)	−0.436	**0.0304**	**0.038**	**0.0885**	0.2656
P-selectin (%) (TRAP-6)	−0.3928	**0.0395**	**0.0439**	**0.0885**	0.3317
PLAs (%) (no agonist)	−0.6045	**0.0017**	**0.008**	**0.0169**	**0.0169**
PLAs (%) (TRAP-6)	−0.5342	**0.0114**	**0.0163**	**0.0452**	0.1083

**Table 2:** The Clinical characteristics of SP MS patients and control group (with reference range); all results are expressed as mean ± SD.

Parameters with Reference Ranges	Control Group (*n* = 45)	SP MS (*n* = 45)
Age	49 ± 11.2	48.6 ± 12.5
Gender (F—Female; M—Male)	28(F); 17(M)	27(F); 18(M)
BMI (kg/m^2^)	22.3 ± 5.1	21.5 ± 9.5
<18.5—underweight		
18.5–24.9—normal weight		
25–29.9—overweight		
30 or greater—obesity [78]		
EDSS (0–10 scale in 0.5 unit increment)	N/A	5.5. ± 1.9
1–4.5—from normal to mid disability		
5–9.5—from moderate to severe disability		
10—death due to MS [79]		
BDI (1–40 scale)	N/A	9.6 ± 4.6
1–9—normal		
10–15—minimal depressive symptomatology		
16–31—mild depression		
32–47—moderate depression		
over 47—severe depression [80]		
Mean disease duration (years)	N/A	14.5 ± 8.1
Blood platelets (130–400 × 10^3^/μL)	258.9 ± 68.10	321.9 ± 43
CRP (<5 mg/L)	3.15 ± 0.9	12.5 ± 5.5

Abbreviations: EDSS—Expanded Disability Status Scale; BDI—Beck’s Depression Inventory; NA—Not Applicable; BMI—Body Mass Index; CRP—C-reactive Protein.

## Abbreviations

**2-DE**	2-dimensional electrophoresis
**ADP**	adenosine diphosphate
**ApoA1**	apolipoprotein A1
**BBB**	blood-brain barrier
**BDI**	Beck’s depression inventory
**BMI**	body mass index
**CIMT**	carotid intima-media thickness
**CIS**	clinically isolated syndrome
**CNS**	central nervous system
**CPDA-1**	citrate phosphate dextrose adenine-1
**CRP**	C-reactive protein
**CSF**	cerebrospinal fluid
**CVD**	cardiovascular disease
**EDSS**	expanded disability status scale
**GPCRs**	G-protein-coupled protease-activated receptors
**GPIIb/IIIa**	glycoprotein IIb/IIIa
**HDL**	high-density lipoprotein
**IgM**	immunoglobulin M
**IL-6**	interleukin-6
**IQR**	interquartile range
**LDL**	low-density lipoprotein
**MALDI-TOF/TOF**	matrix-assisted laser desorption/ionization time-of-flight/time-of-flight
**miRNA**	microRNA
**MMP**	matrix metalloproteinase
**MS**	multiple sclerosis
**PAGs**	platelet aggregates
**PAP**	plasmin–anti-plasmin
**PAR1**	protease-activated receptor 1
**PBMCs**	peripheral blood mononuclear cells
**PF4**	platelet factor 4
**PLAs**	platelet-leukocyte aggregates
**PMPs**	platelet microparticles
**PP MS**	primary progressive multiple sclerosis
**PRP**	platelet-rich plasma
**PSGL-1**	P-selectin glycoprotein ligand 1
**Rho**	Spearman’s rank correlation
**RR MS**	relapsing-remitting multiple sclerosis
**RT-qPCR**	quantitative Real-Time polymerase chain reaction
**SP MS**	secondary progressive multiple sclerosis
**TAT**	thrombin–anti-thrombin
**TNF-α**	tumor necrosis factor α
**TRAP-6**	thrombin receptor-activating peptide 6
**vWF**	von Willebrand factor
**α2M**	α-2-macroglobulin
**β-TG**	β-thromboglobulin
